# *Hosta clausa* (Asparagaceae) in East Asia: Intraspecific chloroplast genome variation and its phylogenomic implications

**DOI:** 10.1371/journal.pone.0317884

**Published:** 2025-02-19

**Authors:** JiYoung Yang, Seung-Chul Kim

**Affiliations:** 1 Research Institute for Dok-do and Ulleung-do Island, Kyungpook National University, Daegu, Republic of Korea; 2 Department of Biological Sciences, Sungkyunkwan University, Suwon, Republic of Korea; G. B. Pant Institute of Himalayan Environment & Development, INDIA

## Abstract

*Hosta* species are abundant in northeastern Asia, offering significant ornamental and horticultural value due to their diverse foliage colors and textures, as well as their showy, fragrant flowers. Among the eight taxa naturally distributed in Korea, *H. clausa* is found in central and northern Korea, as well as northeastern China, providing valuable resources for developing and improving new varieties. Currently, four intraspecific taxa of *H. clausa* are recognized at the variety level based on reproductive (opened vs. closed perianth), vegetative (leaf shape), and habitat characteristics: var. *clausa*, var. *normalis*, var. *ensata*, and var. *geumgangensis*. Despite its horticultural and taxonomic importance, little is known about the degree of intraspecific chloroplast genome variation and relationships among the varieties of *H. clausa*. This could provide some valuable information for marker-assisted breeding programs and molecular cultivar identification. In this study, we investigated the complete plastid genome of 14 accessions of *H. clausa*, covering its native distribution range. We characterized genome size and features and performed comparative plastome analyses (frequency of codon usage, nucleotide diversity, mutation hotspots). Our analysis revealed highly conserved structures and gene content organization in *H*. *clausa*, along with significantly (two to three times) lower nucleotide diversity compared to intraspecific herbaceous and woody species. The phylogenetic analysis did not support the recognition of intraspecific taxa as currently delimited, and a broad-scale geographical structure of complete plastomes was not apparent. The asexual reproductive mode of *H. clausa* appears to contribute to the low plastome genetic diversity. A total of 72 polymorphic sites identified among 14 accessions of *H. clausa* and their phylogenetic relationships, in conjunction with their geographical distribution and morphological characteristics, will be a valuable resource for barcoding study, marker-assisted breeding programs, and developing conservation strategies for hosta species in East Asia.

## Introduction

The genus *Hosta* Tratt. (Asparagaceae) encompasses approximately 40 species of widely popular garden plants primarily distributed throughout northeastern Asia [1–3]. The hypothesized origin of *Hosta* lies along the border between the East China Sea and the East Sea/Sea of Japan, with Japan being considered the center of its species diversity [3]. The most recent classification system of *Hosta* recognizes three subgenera (*Bryocles*, *Giboshi*, and *Hosta*), primarily based on the geographical distribution of the species [3]. The subgenus *Hosta*, which comprises a single species, is restricted to China (*H. plantagenia*), while the other two subgenera, *Bryocles* and *Giboshi*, consist of nine species (found in China and Korea; *H. capitata*, *H. clausa*, *H. ventricosa*, *H. yingeri*, *H. jonesii*, *H. minor*, *H. tibae*, *H. tsushimensis*, and *H. venusta*) and 13 species (found in Japan and the Russian Far East; *H. kiyosumiensis*, *H. sieboldiana*, *H. gracillima*, *H. longissimi*, *H. rectifolia*, *H. sieboldii*, *H. hypoleuca*, *H. longipes*, *H. rupifraga, H. kikutii*, *H. pulchella*, *H. pycnophylla*, and *H. shikokiana*), respectively. Infrageneric classification systems and their interspecific relationships remain highly contentious and necessitate further investigation [1,3–6].

Approximately eight taxa of *Hosta* (six species and two varieties; *H. capitata*, *H. clausa*, *H. venusta*, *H. minor*, *H. yingeri*, and *H. jonesii*; *H. clausa* var. *normalis* and *H. capitata* var. *geumgangensis*) primarily belong to the subg. *Bryocles* and are distributed in Korea [7,8]. Some *Hosta* species tend to have broader distributions; for example, *H. capitata* is mainly found in the central and southern parts of the Korean Peninsula and the Japanese Archipelago, while *H. clausa* occurs in central and northern Korea and northeastern China. *Hosta minor* is endemic to Korea and is found in the central and southern parts of the peninsula. However, certain species are highly restricted to narrower geographical regions; for instance, two Korean endemics, *H. yingeri* and *H. venusta*, are found in the southern part of the Korean Peninsula, including several remote islands. Challenges in species delimitation arise from limited vegetative and reproductive characteristics, frequent hybridization, insufficient comprehensive comparative analysis, and the absence of a robust phylogenetic framework [9]. Recently, Hyb-Seq data [10] have demonstrated strong monophyly of each species, except for *H. minor* and *H. venusta*, and have revealed a sister relationship between *H. minor*/*H. venusta* and *H. jonesii*, as well as between *H. clausa* and cultivated accessions of *H. ventricosa* (which is endemic to China). Furthermore, the earliest divergence of *H. yingeri* within the Korean *Hosta* was inferred, suggesting rapid diversification of Korean *Hosta* during the late Miocene. Lastly, evidence of phylogenomic conflict between the nuclear and chloroplast genomes complicates interspecific evolutionary relationships [10].

*Hosta clausa* is a rhizomatous herbaceous perennial found in central and northern Korea and northeastern China, specifically Liaoning and Jilin Provinces [11]. This species is distinguished from others by several diagnostic features, including clasping ground bracts, flowering bracts that wither to whitish-brown after flowering, dark purple anthers, terete scapes, and elevated veins on the abaxial surface [11]. It primarily inhabits areas along rivers, although its populations tend to be small, isolated, and patchy. The presence of three distinct morphs within the populations complicates infraspecific classification: (1) Maekawa’s *H. ensata* (*H. clausa* var. *ensata*), characterized by lanceolate leaves occurring in streamside rock outcrops (Chinese populations of *H. clausa* var. *normalis* were considered *H. ensata*); (2) *H. clausa* var. *normalis*, which features open flowers and fertile diploid individuals (2*n* =  60) with ovate leaf blades found in sandy soil in open areas; and (3) *H. clausa* var. *clausa*, consisting of sterile triploids with closed flowers [12–14]. An additional type, provisionally referred to as *H. clausa* var. *stolonifera* (nom. nud.), lacks scapes or obligate creeping rhizome propagation and is found under the dense cover of native willows (*Salix*) [3]. While *H. clausa* var. *normalis* is the most prevalent morph, *H. clausa* var. *clausa* has been reported to be extremely rare in natural habitats, with Chung [12] failing to locate any individuals with closed flowers. Jo and Kim [15] identified a small natural population (approximately 50 individuals) of *H. clausa* var. *clausa* in the central Korean Peninsula (Chungcheongbuk-do Province) and also reported a new variety, *H. clausa* var. *geumgangensis*, from a sandy-soil area along the Geumgang River in southern Korea (Jeollabuk-do Province) [8]. Due to its limited geographical distribution in Korea and the small number of individuals (approximately 1,000-1,500), *H. clausa* var. *geumgangensis* is classified as an endangered species (EN) [8]. Chung et al. [16] studied allozymes which revealed low levels of genetic diversity within populations and relatively high levels of population differentiation in *H. clausa*, suggesting that various factors such as habitat preferences, self-compatibility, clonal reproduction, and historical events have influenced the genetic patterns of the species. Although these types have been recognized at the variety level (currently var. *clausa*, var. *normalis*, and var. *geumgangensis* [8]), it is necessary to determine whether each variety forms a monophyletic group and to infer their evolutionary relationships.

The genus *Hosta* is one of economically and medicinally important members of the family Asparagaceae. Numerous *Hosta* species and the cultivars (ca. 6300) are popular garden plants throughout all temperate regions owing to their showy flowers, splendid leaves, and shade tolerance [3]. In addition, some medicinal prosperities, such as antioxidant and anti-inflammatory, make them very valuable in medical areas [17–20]. In addition to population genetic studies primarily based on allozymes [21], several chloroplast genome based studies are available, providing valuable insights into genome organization and evolution, as well as phylogenetic relationships among species within the genus [9,22–24]. While phylogenetic relationships primarily based on a single chloroplast genome per species elucidated interspecific relationships in Korea, we never assessed the genetic variation of chloroplast genomes within a species of *Hosta*. In addition, most recently described taxon in Korea, *H. clausa* var. *geumgangensis*, is unknown for its chloroplast genome feature and its relationship relative to conspecific varieties, *H. clausa* var. *normalis*, and *H. clausa* var. *ensata*. Thus, in this study, we assembled the complete chloroplast genome of *H. clausa* var. *geumgangensis* and compared it those of other conspecific varieties, allowing us to determine the degree of chloroplast genome variation within *H. clausa* and any geographical pattern in Korea. The specific objectives of this study were as follows: (1) to determine the phylogenetic position of the newly described *H. clausa* var. *geumgangensis*; (2) to evaluate the overall relationships among the three varieties; (3) to assess the complete chloroplast genome variation within the species; and (4) to understand intraspecific chloroplast genome evolution.

## Materials and methods

### Plant materials

**Fig 1:**
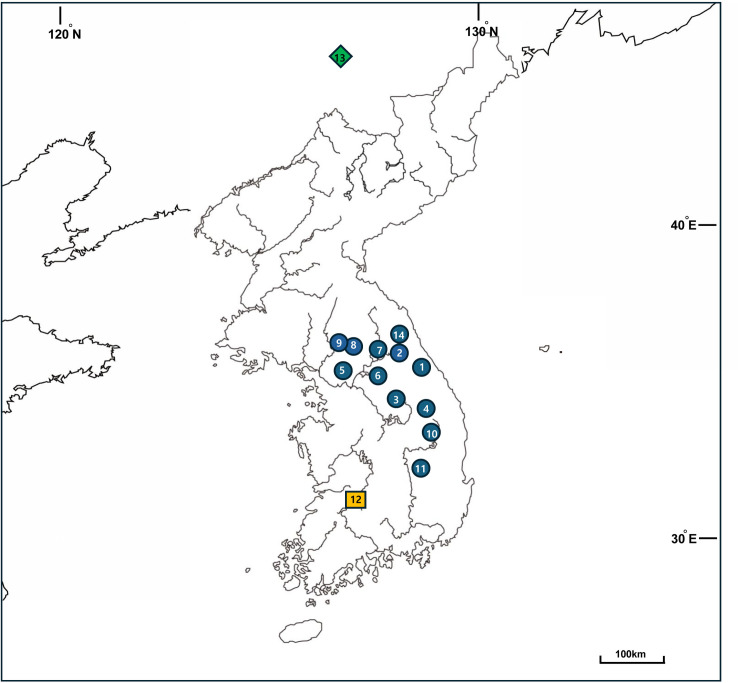
The outline map of South Korea and China depicts the locations where 14 *H. clausa* accessions were collected. The accession numbers correspond to those in Table 1. The map, which is similar but not identical to the original image and is thus for illustrative purpose only, was obtained from the United States Geological Survey (USGS) National Map Viewer (http://viewer.nationalmap.gov/viewer/). Accessed on May 10, 2024.

*H. clausa* var. *geumgangensis* was sampled from its type locality, the Geumgang River in Jinan, Jeollabuk-do Province, Korea (latitude 35.813333°, longitude 127.514220°, and elevation 273 m). No permit was required for collection at this location. A voucher specimen (JL40901) was deposited at the Sungkyunkwan University Ha Eun Herbarium (SKK), Korea. Identification of the plant samples was conducted by Jo Hyun, an expert in *Hosta* species in Korea, and Seung-Chul Kim. For other *H. clausa* accessions, we obtained the complete chloroplast genome sequences from the raw Hyb-Seq data published by Yoo et al. [10]. We did not include the Hyb-Seq data for four cultivars (one accession of ‘Chameleon’, two accessions of ‘Goesan Golden Lightening’, and one accession of ‘Golden Wonder’ from Hantaek Botanical Garden, Korea) because we focused on wild accessions only in this study. Thus, a total of 11 accessions of wild *H. clausa* were selected for analysis (refer to S1 Table of Yoo et al. [10]). The raw chloroplast DNA sequences in FASTA formats (Hosta_cp_v2_fas) of Yoo et al. [10] were downloaded from Figshare (igshare.com/articles/dataset/Phylogenomics_with_Hyb-Seq_Unravels_Korean_Hosta_Evolution/14043965) and cleaned, annotated in order to characterize their complete circular chloroplast genome features (JiYoung Yang, accessed on September 25, 2023). Consequently, we re-analyzed 13 wild accessions with complete and accurate annotations, comprising 12 accessions of *H. clausa* var. *normalis* and one accession of *H. clausa* var. *ensata* (MN901630), sampled from Jilin Province, China. All the wild accessions of *H. clausa* var. *normalis* analyzed were from three provinces in Korea: Gyeonggi-do (GG) (5 accessions: Gapyeong 300097, Pocheon 122790, Pocheon Hantan River 138461, Soyosan 15484, and Yeonchon 0309-1), Gyeongsangbuk-do (GB) (2 accessions: Andong 15327 and Cheongsong 15320), and Gangwon-do (GW) (5 accessions: Cheolwon NC046896, Gangneung 307087, Inje 0828, Jeongseon 15299, and Taebaeksan 464947). These accessions covered the typical geographical distribution of *H. clausa* on the Korean Peninsula (Table 1 and Fig 1).

**Table 1:** Summary of the characteristics of the 14 *H. clausa* chloroplast genomes.

	Taxa	Total cpDNA size (bp)	GC content (%)	LSC size (bp) /GC content (%)	IR size (bp) /GC content (%)	SSC size (bp) /GC content (%)	Number of genes	Number of protein-coding genes	Locality		
1	*H. clausa*_307087	156,622	37.8	84,998/35.9	26,696/42.9	18,232/31.9	132	84	Korea	Gangwon-do (GW)	Gangneung, Jeodong
2	*H. clausa*_0828	156,625	37.8	85,005/35.9	26,696/42.9	18,228/31.9	132	84	Korea		Inje, Mt. Daeam
3	*H. clausa*_15299	156,647	37.8	85,004/35.9	26,695/42.9	18,309/31.9	132	84	Korea		Jeongsun, River Donggang
4	*H. clausa*_464947	156,624	37.8	85,004/35.9	26,696/42.9	18,228/31.9	132	84	Korea		Taebaek, Mt. Taebaek
5	*H. clausa*_15484	156,574	37.8	84,967/35.9	26,696/42.9	18,215/31.9	132	84	Korea	Gyeonggi-do (GG)	Dongduchun, Mt. Soyo
6	*H. clausa*_300097	156,624	37.8	85,004/35.9	26,696/42.9	18,228/31.9	132	84	Korea		Gapyeong, Yeomso-ri
7	*H. clausa*_127790	156,620	37.8	85,005/35.9	26,692/42.9	18,229/31.9	132	84	Korea		Pocheon, Mt. Baekun
8	*H. clausa*_138461	156,622	37.8	84,998/35.9	26,696/42.9	18,232/31.9	132	84	Korea		Pocheon, River Hantan
9	*H. clausa*_0309_1	156,621	37.8	84,998/35.9	26,695/42.9	18,232/31.9	132	84	Korea	Gyeongsangbuk-do (GB)	Yeonchon, Mt. Soyo
10	*H. clausa*_15327	156,461	37.8	84,853/35.9	26,691/42.9	18,220/31.9	132	84	Korea		Andong, Dosan-myeon
11	*H. clausa*_15320	155,514	37.9	84,407/36.0	26,658/42.9	17,784/32.0	132	84	Korea		Cheongsong, Dyukgye-ri
12	*H. clausa* var. *geumgangensis*	156,567	37.8	84,959/35.9	26,697/42.9	18,214/31.9	132	84	Korea	Jeollabuk-do (JB)	Jinan, River Geumgang
13	*H. clausa* var. *ensata* (MN901630)	156,712	37.8	85,092/35.9	26,695/42.9	18,230/31.9	132	84	China	Jinlin	Jilin, Mt. Baishi
14	*H. clausa* var. *normalis* (NC046896)	156,624	37.8	85,004/35.9	26,696/42.9	18,228/31.9	132	84	Korea	Gangwon-do (GW)	Yongneup, Mt. Daeam

### DNA extraction, sequencing, plastome assembly, and annotation

DNA isolation and the generation of complete plastome sequence of newly described Korean endemic *H. clausa* var. *geumgangensis* were performed as described previously [9,25]. In brief, total DNA was extracted using the DNeasy Plant Mini Kit (Qiagen, Carlsbad, CA, USA) and sequenced using an Illumina HiSeq 4000 (Illumina Inc., San Diego, CA, USA), resulting in a 150 bp paired-end read length, at Macrogen Company (Seoul, Korea). The resulting paired-end reads were de novo assembled using Velvet v1.2.10, with multiple k-mers [26]. tRNAscan-SE [27] was employed to confirm the tRNAs, and the sequences were annotated using Geneious R10 [28]. Annotated sequence files in GenBank format were utilized to generate circular maps using OGDRAW v1.2 [29].

### Comparative plastome analysis

We conducted several comparative plastome analyses on 14 accessions of *H. clausa* after aligning them with MAFFT ver. 7 [30], followed by manual adjustments using Geneious R10 [28]. Due to the highly conserved nature of the intraspecific plastomes of *H. clausa*, there were no missing data. Codon usage frequency was calculated using MEGA7 [31] to determine the relative synonymous codon usage (RSCU) value, employing DNA codes utilized by bacteria, archaea, prokaryotic viruses, and chloroplast proteins [32]. The overall sequence divergence was estimated using the LAGAN alignment mode [33] in mVISTA [34]. Subsequently, we performed a sliding window analysis with a step size of 200 bp and a window length of 800 bp using DnaSP 6.10 [35] to ascertain the nucleotide diversity (Pi) of the *H. clausa* plastomes. The aligned cp genomes were then partitioned into the large single-copy region (LSC), the small single-copy region (SSC), and two inverted repeat regions (IRa and IRb). Single nucleotide polymorphism (SNP) sites in each partitioned region were identified to calculate genetic diversity (Pi value and Theta-W) using DnaSP 6.10 [35].

### Phylogenetic analysis

To infer the phylogenetic relationships among the 14 accessions of *H. clausa*, we employed *H. tibae* (MZ919313) as an outgroup [9]. The alignment was conducted using MAFFT ver. 7 [30] in Geneious R10 [28]. Maximum likelihood trees based on both complete plastid genome sequences and concatenated sequences of 82 protein-coding genes were generated with 1,000 replicate bootstrap (BS) analyses using IQ-TREE [36]. The best-fit evolutionary model, “F81+F+I,” was determined using ModelFinder [37] implemented in IQ-TREE. To establish haplotype relationships among the 14 accessions of *H. clausa*, we utilized the haplotype network program TCS version 1.21 [38], treating gaps as missing data. The connection limit, excluding homoplastic changes, was set to 95% following Hart and Sunday [39].

## Results

### Genome size and features

**Fig 2:**
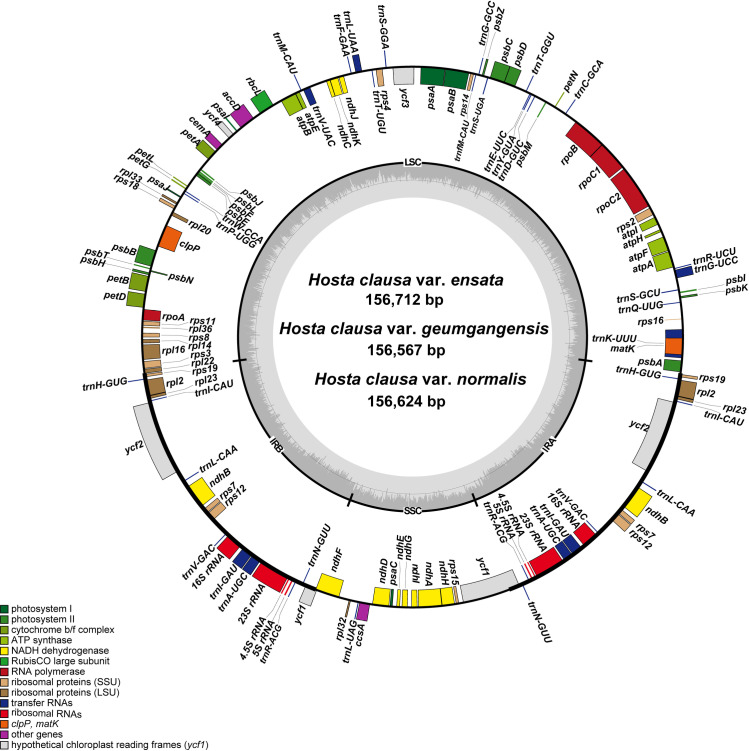
Complete plastome map of the three varieties of *H. clausa* accessions. Genes located outside the circle are transcribed counterclockwise, while those insides are transcribed clockwise.

The complete plastome sequence of *H. clausa* var. *geumgangensis* (GenBank accession number: OL628765) was 156,567 bp, consisting of a large single copy (LSC; 84,959 bp), a small single copy (SSC; 18,214 bp), and two inverted repeat regions (IRa and IRb; 26,697 bp each) (Table 1, Fig 2). The overall GC content was 37.8% (LSC, 35.9%; SSC, 31.9%; IRs, 42.9%). The plastome contained 132 genes, including 84 protein-coding, eight rRNA, and 38 tRNA genes. The partial *ycf1* gene (1,113 bp) was located at the IRb/SSC junction, while the complete *ycf1* gene (5,412 bp) resided in the IR region at the SSC/IRa junction. Notably, one *rps16* pseudogene was situated in the LSC. The complete plastome sequence of *H. clausa* var. *ensata* (MN901630) from China was 156,712 bp, with an LSC of 85,092 bp, an SSC of 18,230 bp, and IRa and IRb regions of 26,695 bp each (Table 1, Fig 2). This plastome harbored 84 protein-coding, eight rRNA, and 38 tRNA genes, identical to var. *geumgangensis*. The remaining 12 plastome sequences of *H. clausa* var. *normalis* ranged from 155,514 bp (Cheongsong, 15320) with an LSC of 84,407 bp, an SSC of 17,784 bp, and an IR of 26,658 bp, to 156,647 bp (Jeongsun, 15299) with an LSC of 85,004 bp, an SSC of 18,309 bp, and an IR of 26,695 bp. The number of protein-coding, rRNA, and tRNA genes remained consistent with var. *ensata*. All plastomes contained 19 duplicated genes in the IR region, comprising eight tRNA, four rRNA, and seven protein-coding genes. Fourteen genes (*ndhA*, *ndhB*, *petB*, *petD*, *rpl2*, *rpl16*, *rpoC1*, *rps12*, *trnA*-UGC, *trnG*-UCC, *trnI*-GAU, *trnK*-UUU, *trnL*-UAA, and *trnV*-UAC) each harbored one intron, while *clpP* and *ycf3* contained two introns. The complete *ycf1* gene was consistently positioned at the SSC/IRa junction across all 14 plastomes, except for *H. clausa* Cheongsong_15320, which had a length of 5,406 bp, and pseudogenized *ycf1* was in the IRb/SSC junction region, except for *H. clausa* Andong_15327, Soyo_15484, and *H. clausa* var. *geumgangensis*. Notably, one *rps16* pseudogene was in the LSC region across all 14 complete plastomes of *H. clausa*.

**Fig 3:**
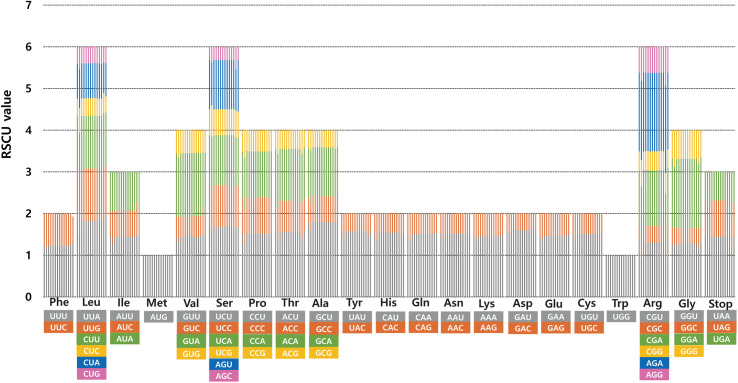
Relative synonymous codon usage (RSCU) in the 14 accessions of *H. clausa*, including three varieties (var. *normalis*, var. *geumgangensis*, and var. *ensata*). The list of accessions from left to right columns represents: 1. *H. clausa* var. *normalis* (NC046896); 2. Andong 15327; 3. Cheongsong 15320; 4. Gangneung 307087; 5. Gapyeong 300097; 6. *H. clausa* var. *geumgangensis*; 7. Pocheon, Hantan River 138461; 8. Inje 0828; 9. Jeonsun 15299; 10. Pocheon Mt. Baekun 122790; 11. Dongduchun Mt. Soyo15484; 12. Taebaek 464947; 13. Yeonchon 0309_1; 14. *H. clausa* var. *ensata* MN901630.

The frequency of codon usage in the 14 accessions of *H. clausa* plastomes was calculated based on the sequences of protein-coding genes. The results revealed that the average codon usage among the 14 accessions ranged from 22,419 (*H. clausa* var. *ensata*) to 26,492 (*H. clausa* var. *normalis*) (S1 Table). The average codon usage for the remaining accessions was 25,301 for the Cheongsong 15320 accession of var. *normalis*, and 26,079 for var. *geumgangensis*, 26,090 for several accessions of var. *normalis* (Dongduchun Mt. Soyo15484, Gangneung 307087, Gapyeong 300097, Pocheon Mountain Baekun 122790, Pocheon Hantan River 138461, and Taebaek 464947), 26,095 for Inje_0828 accession, 26,096 for two accessions (Andong 15327 and Jeongsun 15299), and 26219 for Yeonchon 0309_1 accession. The highest relative synonymous codon usage (RSCU) value was observed in the usage of UUA codon for leucine (1.47–1.83), followed by GCU for alanine (1.61–1.81) and UCU for serine (1.55–1.7). The distribution of codon types was consistent (Fig 3), with codons AUG (M) and UGG (W) encoding methionine and tryptophan, respectively, showing no bias (RSCU = 1) (S1 Table).

### Comparative plastome analyses

**Fig 4:**
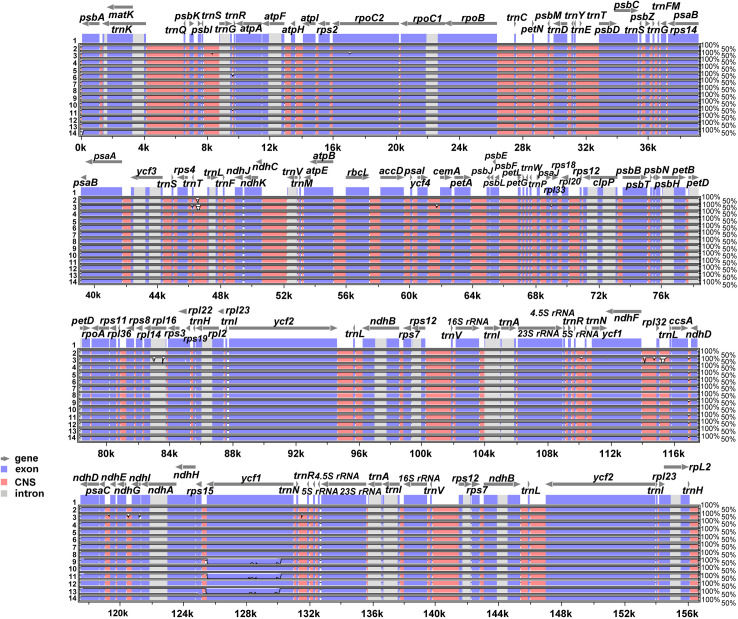
Visualization of the alignment of the 14 plastome sequences of *H. clausa* accessions, including three varieties (var. *normalis*, var. *geumgangensis*, and var. *ensata*). 1. *H. clausa* var. *normalis* (NC046896); 6. *H. clausa* var. *geumgangensis*; 14. *H. clausa* var. *ensata* MN901630; 11 *H. clausa* var. *normalis* accessions (2. Andong 15327; 3. Cheongsong 15320; 4. Gangneung 307087; 5. Gapyeong 300097; 7. Pocheon Hantan River 138461; 8. Inje 0828; 9. Jeonsun 15299; 10. Pocheon Mt. Baekun 122790; 11. Dongduchun Mt. Soyo15484; 12. Taebaek 464947; 13. Yeoncheon 0309_1).

We conducted comparative analyses of three complete plastome sequences from different varieties of *H. clausa*, incorporating 11 additional chloroplast genome sequences of *H. clausa* accessions. These sequences were aligned and analyzed using mVISTA software [34], with the annotated plastome of *H. clausa* var. *normalis* serving as the reference (Fig 4). Our findings indicate a high level of conservation across most regions, with minimal sequence variation observed among the *H. clausa* plastomes. Specifically, we identified 72 polymorphic sites (SNPs) within the 14 chloroplast genomes of *H. clausa*, yielding a Pi value of 0.00015 and a Theta-W value of 0.00015 (Table 2). Notably, the SSC region exhibited the highest polymorphism, with 21 SNPs and Pi and Theta-W values of 0.00046 and 0.00037, respectively, while the LSC region displayed relatively lower polymorphism, detecting 49 SNPs with Pi and Theta-W values of 0.00018 each. The IR region showed significantly lower polymorphism, with only one SNP and Pi and Theta-W values of 0.00001 each. Furthermore, the non-coding region of the chloroplast genome in *H. clausa* spanned a total length of 78,109 bp, with 45 SNPs and Pi and Theta-W values of 0.0002 and 0.00018, respectively. Conversely, the coding region encompassed a total length of 78,608 bp, with 27 SNPs and identical Pi and Theta-W values of 0.00012 (Table 2).

**Table 2:** Nucleotide diversity (Pi, Theta-W) of the 14 *H. clausa* accessions.

	Genome	LSC	SSC	IRa	Noncoding Region	Coding Region
Total number of sites	156,717	85,046	18,253	26,709	78,109	78,608
Number of polymorphic sites	72	49	21	1	45	27
Pi	0.00015	0.00018	0.00046	0.00001	0.0002	0.00012
Theta-W	0.00015	0.00018	0.00037	0.00001	0.00018	0.00012

**Fig 5:**
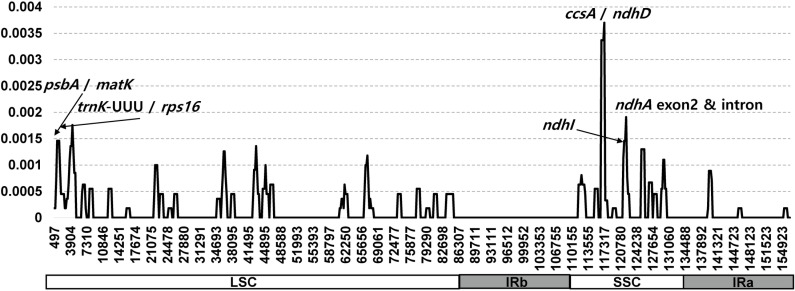
Sliding window analysis of the 14 whole chloroplast genomes of *H. clausa* accessions.

Additionally, sliding window analysis identified five highly variable regions within the 14 plastomes of *H. clausa* (Fig 5). The average nucleotide diversity (Pi) across the entire cp genome was calculated as 0.000159. Notably, the *ccsA*/*ndhD* intergenic region exhibited the highest variability, with a Pi value of 0.0037. Furthermore, four other regions demonstrated significant variability: *ndhA* exon2 and intron (Pi =  0.00191), *trnK*-UUU/*rps16* (Pi =  0.00176), *psaA*/*matK* (Pi =  0.00146), and *ndhI* (Pi =  0.00146).

### Plastome phylogenomic and network analyses of *H. clausa* accessions

**Fig 6:**
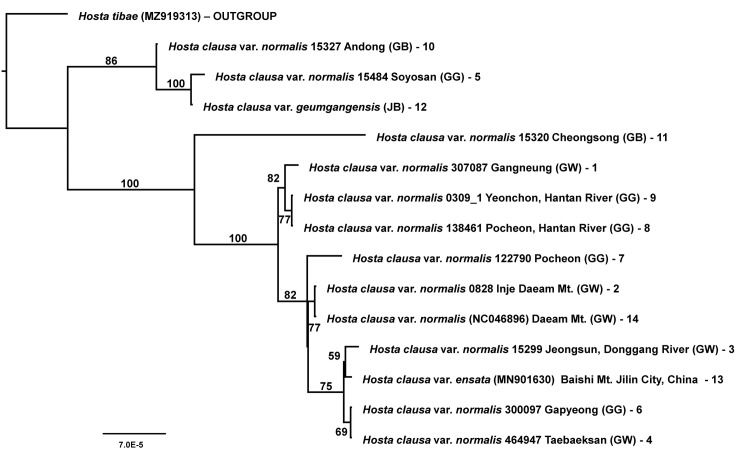
The maximum-likelihood (ML) tree based on the 14 accessions of chloroplast genomes of *H. clausa*, including var. *normalis* (12 accessions), var. *ensata* (one accession), and var. *geumgangensis* (one accession). *Hosta tibae* was selected as an outgroup. The bootstrap value based on 1,000 replicates is shown on each node.

The maximum likelihood tree revealed three major lineages within *H. clausa* (Fig 6). The first lineage (86% bootstrap support, BS) included three accessions: var. *geumgangensis* (Jeollabuk-do) and two accessions of var. *clausa* (15327 Andong, GB and 15484 Soyosan, GG). These three accessions were geographically widely separated from each other, distributed in the northwest (15484, GG), southwest (var. *geumgangensis*), and the central southeast (15327, GB). The second lineage included a single accession sampled from the central southeast (15320 Cheongsong, GB), which was geographically close to accession 15327 (Andong, GB). The third lineage (82% BS) included three accessions primarily from northern areas, including two accessions from Gyeonggi-do, GG (Yeonchon 0309_1 and Pocheon Hantan River 138461) and one accession from Gangwon-do, GW (Gangneung 307087). The last lineage (82% BS) included the remaining accessions, primarily from the central Korean Peninsula (GW and GG), and included var. *ensata* (=*H. ensata*) from northeastern China. Overall, the maximum likelihood tree-based phylogenetic relationships among accessions of *H. clausa* revealed no clear broad-scale geographical plastome patterns in the Korean Peninsula. However, in some cases, geographically closer populations (Inje 0828 and NC046896 from Daeamsan Mountain) or from the same river system (0309_1 and 138461 from the Hantan River) formed a clade, indicating a small-scale geographical pattern.

**Fig 7:**
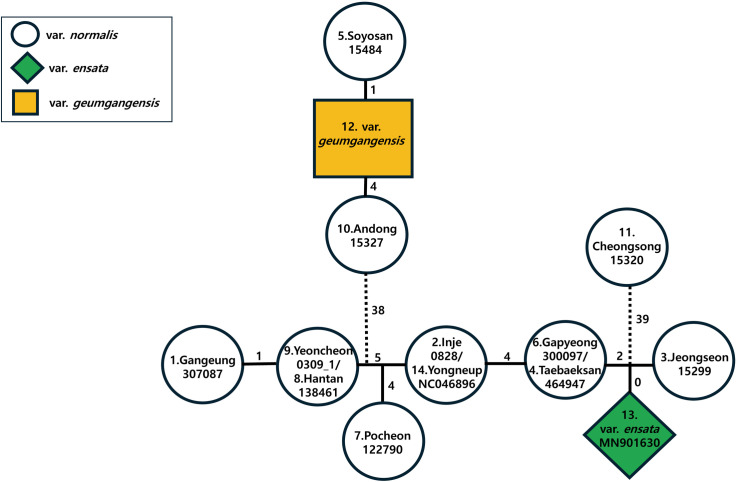
TCS network of the 11 chloroplast haplotypes found in *H. clausa* whole plastomes in this study. The number of missing or inferred haplotypes is indicated as numerical values between haplotypes.

The complete plastome haplotype network was determined using TCS analysis (Fig 7), further revealing similar haplotype relationships as shown based on the ML tree. Overall, very few inferred (missing) haplotypes, normally fewer than 5, connected the 11 major haplotypes found among the 14 *H. clausa* accessions. However, 38 and 39 inferred haplotypes were required to connect var. *normalis* (Andong 15327 and Soyosan 15484), and var. *geumgangensis* and var. *normalis* (Cheongsong 15320), respectively, to the remaining main haplotypes of var. *normalis*. The var. *geumgangensis* haplotype was closely connected to the Soyosan accession (15484) by one inferred haplotype, while the var. *ensata* haplotype was closely connected to the Jeongseon accession (15299) through two inferred haplotypes.

## Discussion

### Taxonomic implications of complete plastomes of *H. clausa*

The main objective of this study was to determine the phylogenetic position of two varieties of *H. clausa*: var. *ensata* in northeastern China and var. *geumgangensis* in southern Korea. *Hosta clausa* var. *clausa* exhibits closed flowers and seedless capsules, whereas var. *normalis* F. Maekawa has open flowers and capsules with seeds [1,3,8,40–42]. *Hosta clausa* var. *ensata* (F. Maekawa) W.G. Schmid is considered a synonym for *H. clausa* var. *normalis* [42]. Var. *clausa* was not included in this study due to its rarity. Therefore, we evaluated the phylogenetic position of the three remaining varieties: var. *normalis*, var. *ensata*, and var. *geumgangensis*. However, limited sampling of var. *ensata* and var. *geumgangensis* prevented a full assessment of the monophyly of each variety. Nevertheless, our analysis revealed that both varieties were deeply nested within var. *normalis* (Figs 6 and 7). It is plausible that with more extensive sampling, each variety may exhibit monophyly, as seen in small-scale geographical sampling of var. *normalis* (e.g., 0309_1 and 138461; 0828 and NC046896). Nonetheless, var. *geumgangensis* was found to be sister to *H. clausa* var. *normalis* (15484) from the Soyosan Mountains in Central Korea, while var. *ensata* is sister to *H. clausa* var. *normalis* (15299) from the Donggang River. These plastome-based relationships did not appear to align with geographical distribution. However, the ML tree based solely on the CDS (not shown) suggested that var. *geumgangensis* was sister to 15327 (Andong, Gyeongsanbuk-do), a geographically proximate population (Fig 1). Var. *geumgangensis* can be distinguished from other varieties by qualitative traits (open vs. closed flower, seeded vs. seedless capsule, petiole color) and quantitative traits (pistil length and perianth tube length/width) [8]. Due to its geographical isolation from typical var. *normalis* populations in Korea, var. *geumgangensis* is likely reproductively isolated, maintaining a distinct lineage and evolutionary trajectory [8,15]. Similarly, the closed-flower variety *clausa* is likely reproductively isolated from the open-flowered var. *normalis* [15]; however, Chung [12] considered that var. *clausa* and var. *normalis* are biologically one and the same species. These currently recognized varieties [15] likely originated independently from the more widespread var. *normalis*, as indicated by the current study and the phylogenetic relationships between the open-flowered seedless var. *geumgangensis* and closed-flowered var. *clausa* (Figs 6 and 7). Population-level genetic studies, coupled with extensive sampling of the three varieties and reproductive biology investigations, could elucidate their evolutionary origins, ultimately contributing to their taxonomic classification. Previous studies have demonstrated low levels of gene flow among small isolated populations of *H. clausa* on the Korean Peninsula based on allozymes [16], potentially justifying recognition of certain populations at the varietal level with distinct morphological characteristics. However, comprehensive morphological and molecular analyses, especially based on the genome-wide genetic variation, are needed to rigorously test these hypotheses, as exemplified in the study of Yahara et al. [43].

### Intraspecific plastome variation and evolution in *H. clausa*

For the first time, we investigated the overall variation in the complete plastomes of *H. clausa* on the Korean Peninsula. Based on allozymes, it has been suggested that *H. clausa*, which typically occurs along stream sides, maintains low levels of genetic variation and exhibits high levels of population differentiation compared to other species with similar life history traits [16]. We, based on the complete chloroplast genomes, found that the genetic diversity level of the chloroplast genome in *H. clausa*, i.e., Pi =  0.000159), is substantially low compared to other herbaceous (e.g., *Ricinus communis*, 20 wild and cultivated accessions, Pi =  0.00046, Muraguri et al. [44]; *Aegilops tauschii*, 17 accessions, noncoding regions average of 0.00133 and coding regions average of 0.000432, Su et al. [45]) and woody species (e.g., *Styphnolobium japonicum*, 12 accessions, Pi =  0.00029, Mu et al. [46]; *Quercus acutissima*, 3 accessions, Pi =  0.00035, Zhang et al. [47]). The nucleotide diversity (Pi) of the noncoding and coding regions of *H. clausa* (0.0002 and 0.00012, respectively) was much lower than that of *Aegilops tauschii* (0.00133 and 0.000432, respectively) and *S. japonicum* (Pi =  0.00043 and 0.00019, respectively). In addition, the SSC region with the highest nucleotide diversity in *H. clausa* (0.00046) was significantly less diverse than that in *S. japonicum* (0.00085). However, nucleotide diversity in the coding region of *H. clausa* (0.00012) was slightly lower than that of *S. japonicum* (0.00019). Therefore, regardless of the life-history traits of *Hosta* (herbaceous perennials), *Aegilops* (annuals), *Ricinus* (herbaceous perennials), and *Styphnolobium* (woody perennials), it is apparent that *H. clausa* has very low genetic diversity. The geographical sampling of *H. clausa* populations covered the natural distribution areas of the species in the Korean Peninsula in this study, including the northernmost *H. clausa* var. *ensata* from China and most of the southwestern *H. clausa* var. *geumgangensis* from South Korea. It is uncertain why *H. clausa* maintains low genetic diversity in its plastomes, but it is plausible that the sexual and asexual reproductive modes of the species are likely contributing factors. Given its distribution patterns in the Korean Peninsula and northeastern China, *H. clausa* could be a good model system to assess the impact of glaciation in East Asia, testing the “southern richness to northern purity” paradigm [48].

## Conclusions

We assembled the first complete chloroplast genome of recently described species, *H. clausa* var. *geumgangensis* from southern Korea. We then characterized complete chloroplast genome size and feature among 13 accessions of *H. clausa*, including two varieties *geumgangensis* and *ensata*, to investigate intraspecific variation. Our analyses revealed highly conserved structures and gene content organization in *H*. *clausa*, along with significantly lower nucleotide diversity compared to intraspecific herbaceous and woody species. The phylogenetic analysis did not support the recognition of intraspecific taxa as currently delimited, and a broad-scale geographical structure of complete plastomes was not apparent. Additional study based on comprehensive morphological and molecular analyses is required to further assess intraspecific variation and relationship within *H. clausa* in Korea. Seventy-two polymorphic sites identified in this study will be a valuable resource for barcoding and marker-assisted breeding programs for *H. clausa* in Korea.

## Supporting information

S1 Table Codon usage and codon–anticodon recognition pattern for tRNA in the 14 wild *H. clausa* plastomes. Four cultivated accessions from the Hyb-Seq data published by Yoo et al. [10] in Figshare (igshare.com/articles/dataset/Phylogenomics_with_Hyb-Seq_Unravels_Korean_Hosta_Evolution/14043965) were not included due to the main focus of wild accessions only in this study (JiYoung Yang, accessed on September 25, 2023).
(XLSX)
